# Supplementary material for: Pathogen-specific social immunity is associated with erosion of individual immune function in an ant
Source: Nat Commun. 2024 Oct 26;15:9260. doi: 10.1038/s41467-024-53527-4 (PMC11513022; doi:10.1038/s41467-024-53527-4)
Supplement: Supplementary file 1 — Supplementary Information [file 41467_2024_53527_MOESM1_ESM.pdf]

Pathogen-specific social immunity is associated with erosion of individual immune function in an ant

Florent Masson[1*], Rachael Louise Brown[1], Joel Vizueta[2], Thea C. T. Irvine[1], Zijun Xiong[3], Jonathan Romiguier[4], Nathalie Stroeymeyt[1*]

[1] School of Biological Sciences, University of Bristol, Bristol, UK

[2] Villum Centre for Biodiversity Genomics, Section for Ecology and Evolution, Department of Biology, University of Copenhagen, Universitetsparken 15, 2100, Copenhagen, Denmark

[3] BGI Research, Wuhan 430074, China

[4] ISEM, University of Montpellier, CNRS, IRD, CIRAD, Montpellier, France

Table of contents

Supplementary Figures 1 to 6

Supplementary Tables 1 to 4

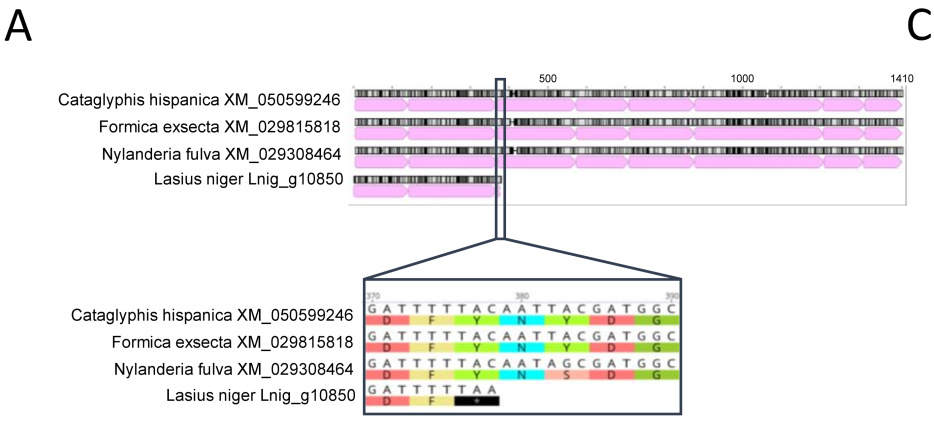

C

Species	Subfamily
Formicidae	
GAGA-0177 Lasius niger	Formicinae
GAGA-0245 Monomorium pharaonis	Myrmicinae
GAGA-0306 Stictoponera bicolor	Ectatomminae
GAGA-0343 Pseudomyrmex spinicola	Pseudomyrmecinae
GAGA-0391 Stigmatomma sp.	Amblyoponinae
GAGA-0392 Leptanilla sp.	Leptanillinae
GAGA-0404 Mystrium camillae	Amblyoponinae
GAGA-0522 Myrmecia croslandi	Myrmeciinae
GAGA-0535 Discothyrea kamiteta	Proceratiinae
GAGA-0552 Paraponera clavata	Paraponerinae
NCBI-0001 Ooceraea biroi	Dorylinae
NCBI-0002 Solenopsis invicta	Myrmicinae
NCBI-0005 Camponotus floridanus	Formicinae
NCBI-0007 Dinoponera quadriceps	Ponerinae
NCBI-0009 Harpegnathos saltator	Ponerinae
NCBI-0010 Linepithema humile	Dolichoderinae
NCBI-0012 Pogonomyrmex barbatus	Myrmicinae
Other Hymenoptera	
Amel – Apis mellifera - Apoidea	
OUTG-0007 - Nysson_spinosus - Apoidea	
OUTG-0008 – Ampulex compressa - Apoidea	
Vcra_Vespoidea – Vespa crabro	
Anoplius_nigerrimus_Pompiloidea – Anoplius nigerrimus	
Nvit_Chalcidoidea – Nasonia vitripenis	
Orussus_abietinus_Orossoidea – Orossus abietinus	
Tenthredo_mesomela_Tenthredinoidea	
Outgroups	
Tcas – Tribolium castaneum	
Dmel – Drosophila melanogaster	

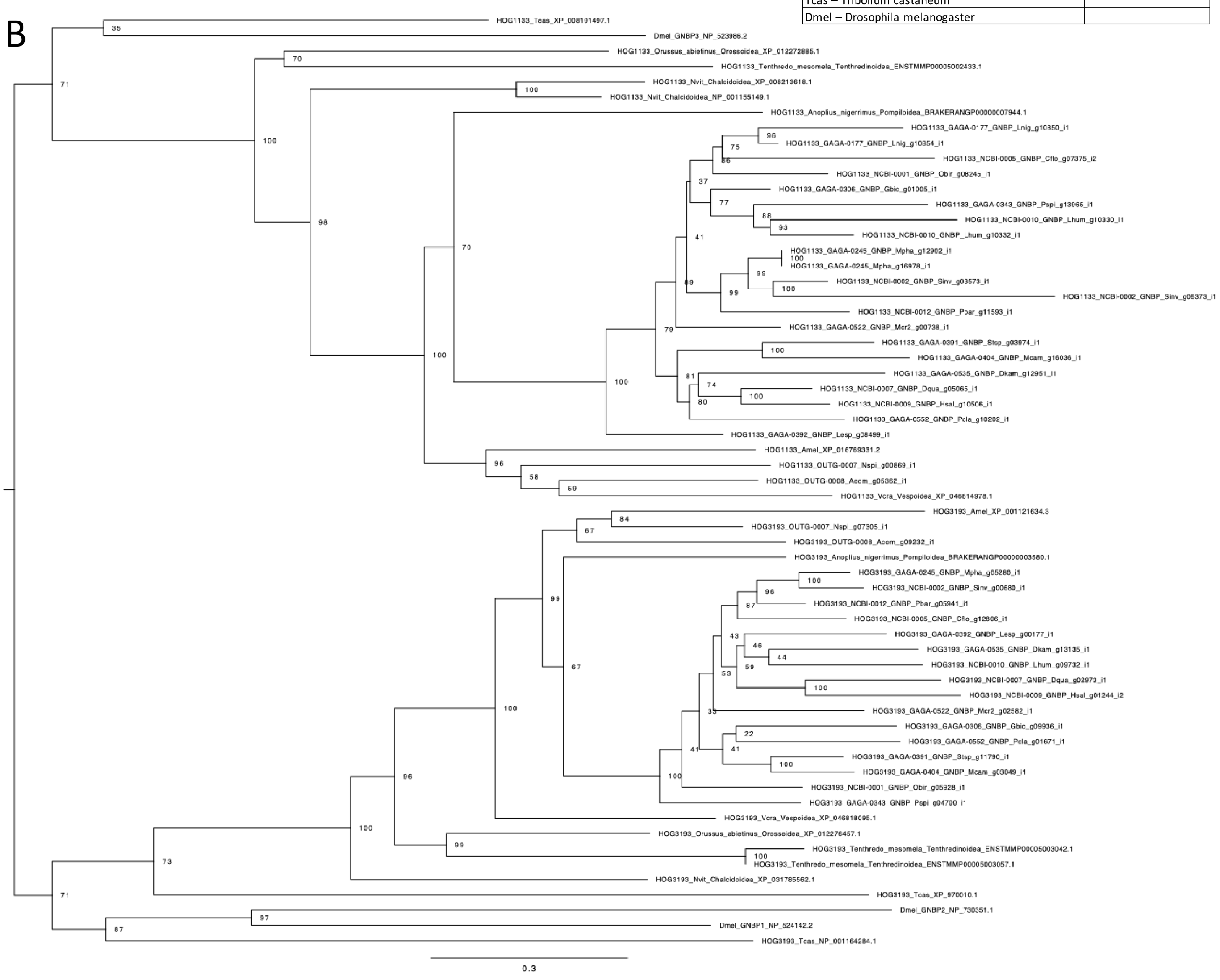

Supplementary Figure 1. The genome of *L. niger* codes for a reduced number of functional GNBP proteins

(A) MAFFT L-INS-I alignment of the GNBP coding sequence of selected Formicinae species. Species name and Genbank accession number are indicated before each sequence. For each sequence the barcode indicates position conservation across species (lightgrey: identical nucleotide in all five species; black: position is different from the consensus sequence in at least one species) and the pink arrows delineate exons. *L. niger* exon 3 is truncated by a premature stop codon visible on the higher magnification inset, where black letters indicate the coding sequence and colored letters the corresponding amino-acid (single-letter code) or stop codon (*). (B) Maximum likelihood gene tree of GNBP coding genes from 17 ant genomes and 8 additional Hymenopteran genomes. *D. melanogaster* and *T. castaneum* were used as outgroups. Other species names are coded with the first letter of the genus name and three first letters of the species name as listed in (C). “g00000” are accession numbers of GAGA genomes for which sequences can be obtaine upon request to the corresponding authors. Other accession numbers refer to publicly available sequences on Genbank. Node labels indicate bootstrap support.

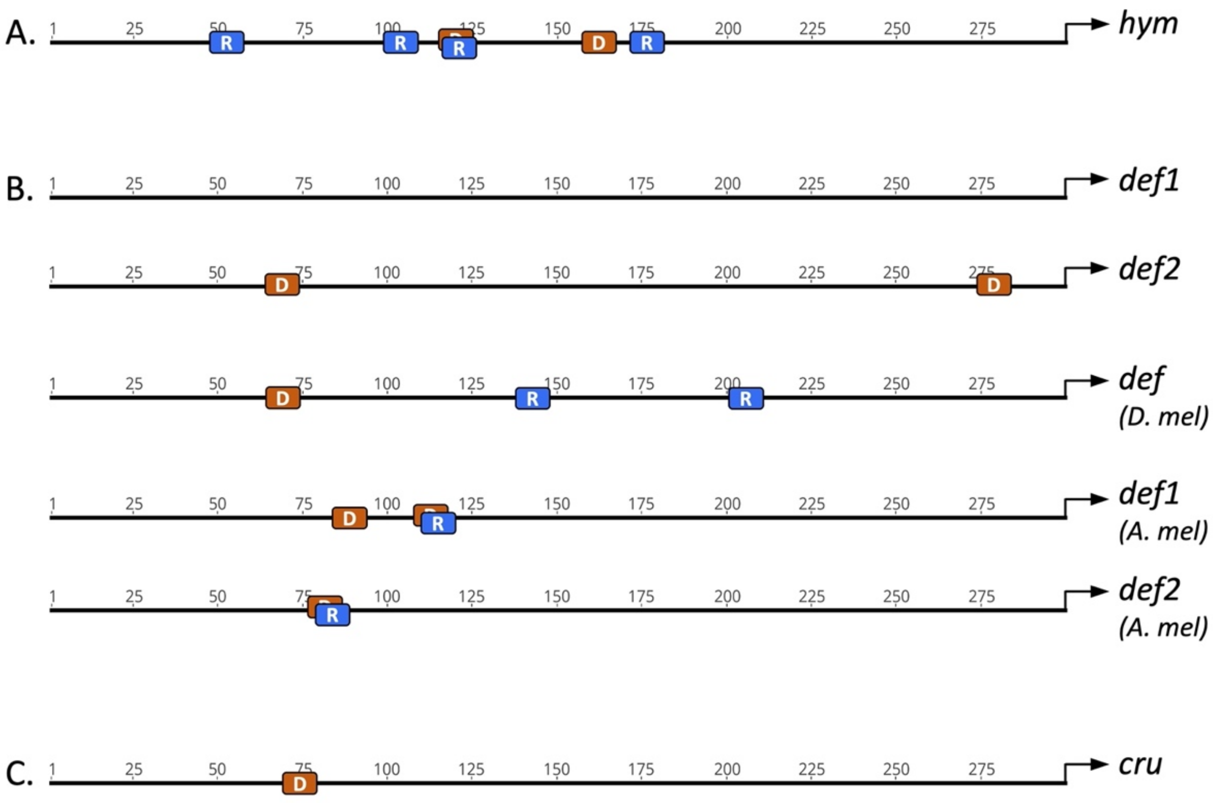

Supplementary Figure 2. Prediction of the transcriptional regulation of *L. niger* AMP coding genes

Predicted NF-κB transcription factor binding sites on the upstream sequences of (A) *hymenoptaecin*, (B) *defensins* and (C) *crustin*. “R” blue boxes indicate predicted Relish-binding sites (IMD regulation) and “D” orange boxes indicate predicted Dorsal-binding sites (Toll regulation). Panel (B) also shows the promoters of *D. melanogaster* (*D. mel*) *defensin* and *A. mellifera* (*A. mel*) *defensin-1* and *defensin-2*.

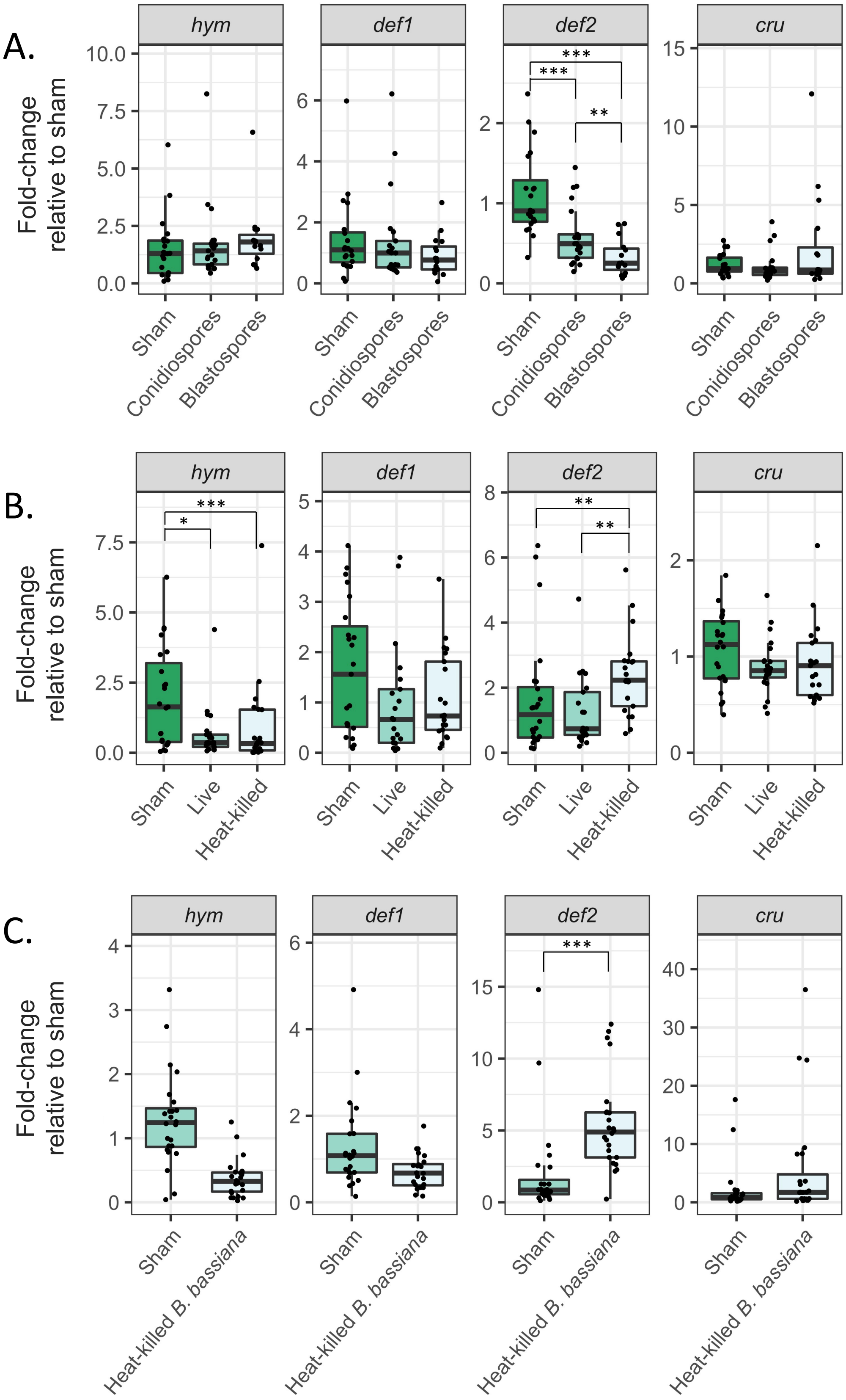
A.
B.
C.
Fold-change relative to sham
hym
def1
def2
cru
Sham
Conidiospores
Blastospores
Live
Heat-killed
Heat-killed B. bassiana
***
**
*

Supplementary Figure 3. The absence of NF-κB response to systemic fungal infections is independent of fungal species, form, or immune evasion mechanisms

(A) Induction of AMP coding genes in response to systemic injections of (A) *M. brunneum* blastospores; (B) heat-killed *M. brunneum* conidiospores; (C) heat-killed *B. bassiana* conidiospores. Boxplots represent the median and interquartile range. The upper/lower whiskers are the values within 1.5 times the interquartile range over/under the 75th percentile. Each dot represents a biological replicate. Data were analyzed by LMM followed by Anova and post-hoc contrasts with Benjamini-Hochberg correction when relevant. ***: $p < 0.001$; **: $p < 0.01$; *: $p < 0.05$. Source data are provided as a Source Data file.

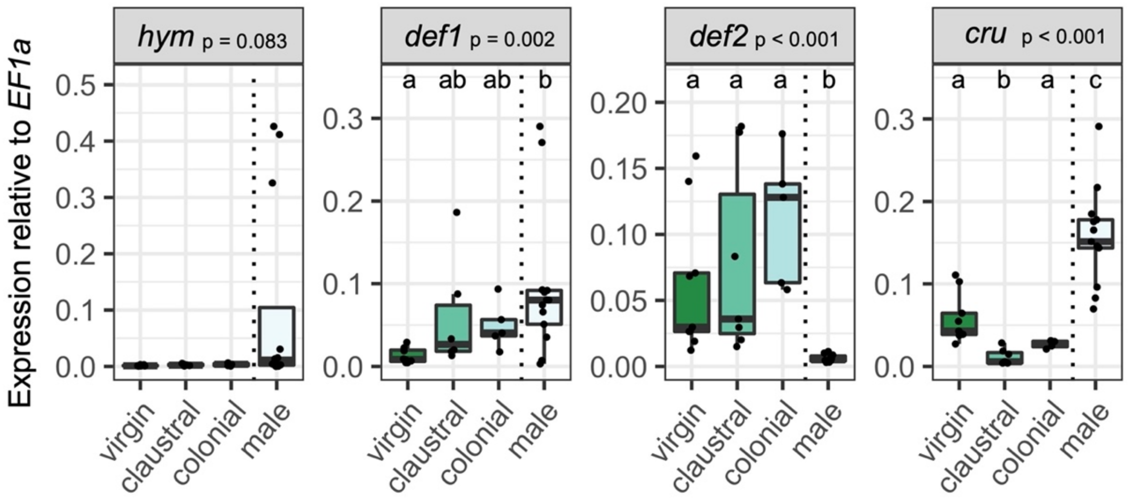

Supplementary Figure 4. Individual immune effector are differentially expressed in non-infected reproductive individuals

RT-qPCR measurement of the basal expression level of AMP coding genes relative to the house-keeping gene *EF1a* in unchallenged reproductive individuals (virgin, claustral and colonial queens and males ; N = 5-8). Boxplots represent the median and interquartile range of biological replicates. The upper/lower whiskers are the values within 1.5 times the interquartile range over/under the 75th percentile. Data were analyzed by LMM. Anova p-values are provided next to each gene name. Letters identifies groups that are statistically different based on post-hoc contrasts with BH correction. Source data are provided as a Source Data file.

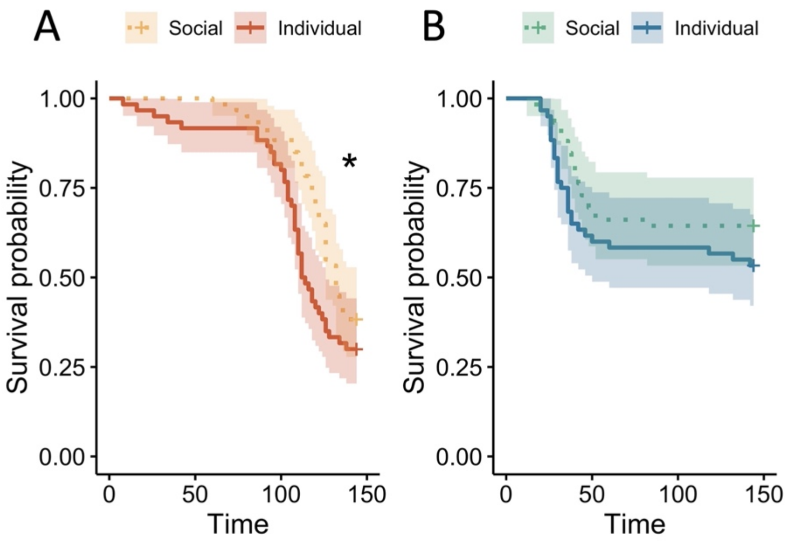

Supplementary Figure 5. Social incubation confers protection against the fungus *B. bassiana* but not the bacterium Ecc15.

Survival of injured ants exposed to (A) *B. bassiana* or (B) Ecc15 when kept socially or in isolation (N = 60). Shading indicates confidence intervals. Data were analyzed by Anova on a mixed-effect Cox model. *: $p < 0.05$. Source data are provided as a Source Data file.

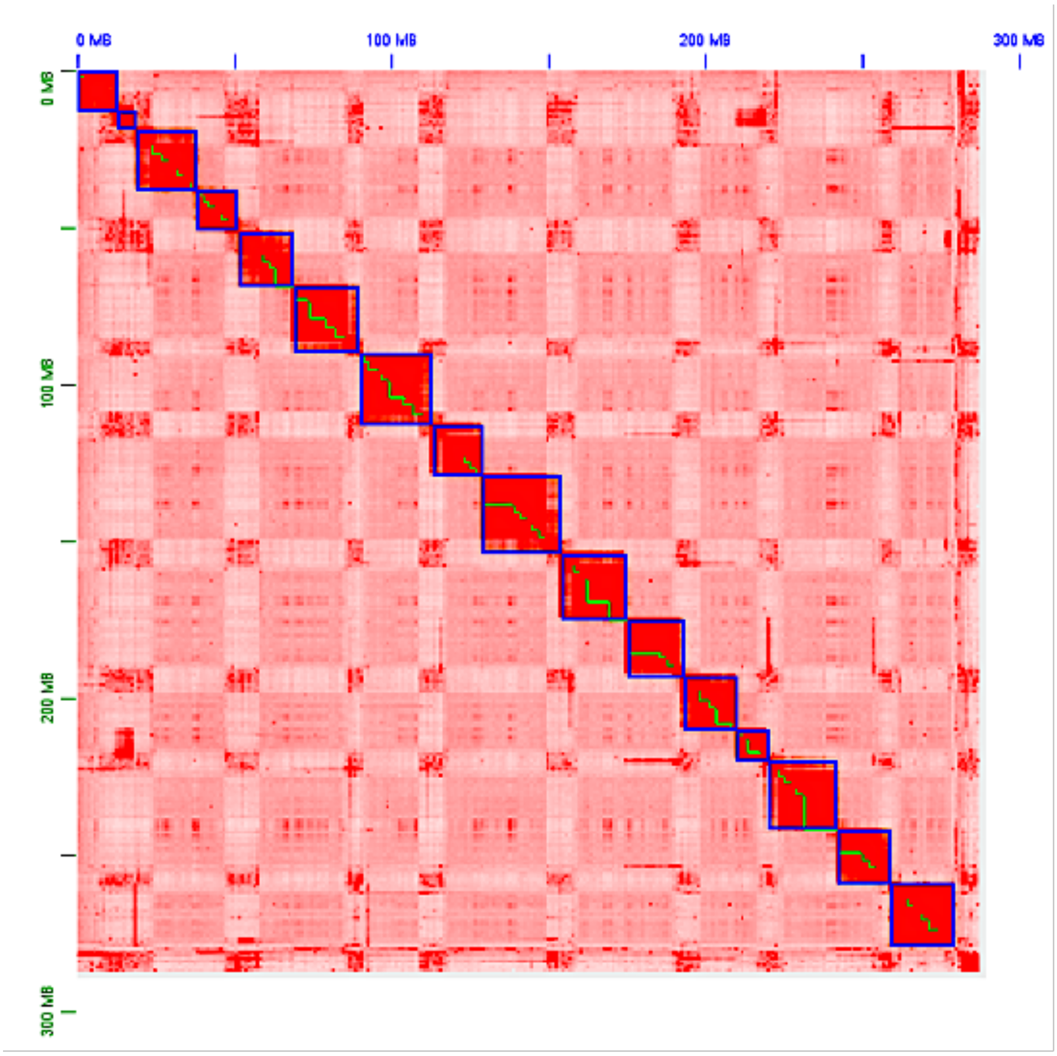

Supplementary Figure 6. Hi-C contact heatmap of the *L. niger* genome. Each block represents the chromatin interactions between two genomic loci. Darker red color indicates higher contact density.

Supplementary Table 1. ***L. niger*** **IMD pathway gene list.**

	**Name**	**Symbol**	**Locus tag**	**BLAST e-value vs *D. melanogaster* homologue**
**Key components**	Peptidoglycan recognition protein LC	PGRP-LC	Lnig_g10065	7.97E-58
	immune deficiency	imd	Lnig_g11799	7.00E-17
	Death-associated inhibitor of apoptosis 2	Diap2	Lnig_g02092	2.34E-84
	TGF-beta activated kinase 1	Tak1	Lnig_g03568	7.74E-105
	TAK1-associated binding protein 2	Tab2	Lnig_g11988	4.00E-04
	Fas-associated death domain	Fadd	Lnig_g03414	8.00E-14
	Death related ced-3/Nedd2-like caspase	Dredd	Lnig_g08104	1.87E-37
	I-kappaB kinase beta	IKKβ (Ird5)	Lnig_g00499	8.19E-42
	I-kappaB kinase gamma	IKKγ (kenny)	Lnig_g15535	2.71E-2, uncertain
	Relish	Rel	Lnig_g04430	3.69E-99
**Secondary components**	akirin	akirin	Lnig_g18272	6.08E-42
	caspar	casp	Lnig_g13315	4.13E-150
	Cullin 1	Cul1	Lnig_g12307	0
	defense repressor 1	dnr1	Lnig_g06056	5.07E-68
	Death related ICE-like caspase	Drice	Lnig_g14613	4.63E-120
	effete	eff	Lnig_g18319	2.35E-109
	falafel	flfl	Lnig_g03390	0
	kayak	kay	Lnig_g12954	2.65E-21
	longitudinals lacking	lola	Lnig_g10405	3.40E-71
	Linear Ubiquitin E3 ligase	LUBEL	Lnig_g03464	7.91E-173
	lesswright	lwr	Lnig_g01816	1.64E-100
	Poly-(ADP-ribose) polymerase	Parp	Lnig_g03489	0
	Peptidoglycan recognition protein LB	PGRP-LB	Lnig_g10175	1.34E-44
	Peptidoglycan recognition protein SC2	PGRP-SC2	Lnig_g10176	1.79E-51
	Protein phosphatase 19C	Pp4-19C	Lnig_g14471	0

	Protein phosphatase 4 regulatory subunit 2-related protein	PPP4R2r	Lnig_g01600	1.67E-51
	scrawny	scny	Lnig_g09033	1.43E-130
	sickie	sick	Lnig_g15056	0
	SKP1-related A	SkpA	Lnig_g14970	1.68E-90
	Suppressor of variegation 2-10	Su(var)2-10	Lnig_g12653	7.02E-154
	TNF-receptor-associated factor 6	Traf6	Lnig_g13057	3.96E-69
	trabid	trbd	Lnig_g00077	0
	Ubiquitin conjugating enzyme 4	Ubc4	Lnig_g10204	4.64E-104
	NEDD8-conjugating enzyme UbcE2M	UbcE2M	Lnig_g15542	2.83E-107
	Ubiquitin-conjugating enzyme variant 1A	Uev1A	Lnig_g03301	3.11E-83
	veloren	velo	Lnig_g01005	3.44E-128

Supplementary Table 2. *L. niger* Toll pathway gene list.

	Name	Symbol	Locus tag	BLAST e-value vs *D. melanogaster* homologue
**Key components**	Gram-negative bacteria binding protein	GNBP	Lnig_g10854	2.37E-68
	Peptidoglycan recognition protein SA	PGRP-SA	Lnig_g03384	4.47E-54
	modular serine protease	modSP	Lnig_g18714	1.49E-48
	spaetzle	spz	Lnig_g05108, Lnig_g14788	N/A
	Toll	Toll	Lnig_g12674	0
	Myd88	Myd88	Lnig_g12877	3.11E-36
	pelle	pll	Lnig_g15441	7.78E-91
	tube	tub	Lnig_g04361	4.00E-12
	cactus	cact	Lnig_g18204	1.80E-55
	dorsal	dl	Lnig_g05807	1.22E-158
**Secondary components**	26-29kD-proteinase	26-29-p	Lnig_g08521	0
	cactin	cactin	Lnig_g12215	0
	deltex	dx	Lnig_g14590	1.13E-81
	foraging	for	Lnig_g04154	0
	G protein-coupled receptor kinase 2	Gprk2	Lnig_g00563	0
	groucho	gro	Lnig_g06093	0
	HECT and RLD domain containing E3 ubiquitin ligase 4	Herc4	Lnig_g08758	0
	kurtz	krz	Lnig_g05010	0
	lesswright	lwr	Lnig_g01816	1.64E-100
	microtubule star	mts	Lnig_g13079	0
	necrotic	nec	Lnig_g11932	1.96E-54
	pipe	pip	Lnig_g03011	1.81E-179
	Pitslre	Pitslre	Lnig_g01484	0
	Protein phosphatase 2A at 29B	Pp2A-29B	Lnig_g08394	0

	Small ubiquitin like modifier	Sumo	Lnig_g11651	5.69E-54
	twins	tws	Lnig_g00415	0
	Ubiquitin-like activating enzyme 2	Uba2	Lnig_g05188	0
	Ulp1	Ulp1	Lnig_g13237	3.16E-68

Supplementary Table 3. ***L. niger*** **antimicrobial peptide coding gene list.**

Name	Symbol	Locus tag
Hymenoptaecin	hym	Lnig_g01772
Defensin 1	def1	Lnig_g14806
Defensin 2	def2	Lnig_g15487
Crustin	cru	Lnig_g00276
Waprin	wap	Lnig_g03675

Supplementary Table 4. ***L. niger*** **genome quality data.**

**Genome size**	287,390,509
**Number of scaffolds**	696
**Scaffold N50**	19,672,953
**Contig N50**	4,062,532
**Longest scaffold**	24,881,522
**L50**	7
**L90**	14
**GC %**	36.71
**BUSCO Hymenoptera (-genome)**	C:97.2%[S:96.9%,D:0.3%],F:0.7%,M:2.1%,n:5991
**Compleasm (BUSCO Hymenoptera dataset)**	S:97.60; D:0.28; F:0.77; I:0.00; M:1.35; N:5991
**Merqury Completeness**	92.8585
**Merqury QV**	34.1226
**Total repeats**	33.44%
**Repeats: DNA elements**	11.35%
**Repeats: LINE**	4.14%
**Repeats: SINE**	0.10%
**Repeats: LTR**	13.65%
**Repeats: Unknown**	11.10%
