# Supplementary material for: Pathogen-specific social immunity is associated with erosion of individual immune function in an ant
Source: Nat Commun. 2024 Oct 26;15:9260. doi: 10.1038/s41467-024-53527-4 (PMC11513022; doi:10.1038/s41467-024-53527-4)
Supplement: Supplementary file 2 — Description of Additional Supplementary Files [file 41467_2024_53527_MOESM2_ESM.pdf]

Description of Additional Supplementary Files

Supplementary Movie 1. Slow-motion illustration of a fight between *M. rubra* and *L. niger.*
